# RNA-Binding Proteins: A Role in Neurotoxicity?

**DOI:** 10.1007/s12640-023-00669-w

**Published:** 2023-09-30

**Authors:** Andrea Ocharán-Mercado, Jaqueline Loaeza-Loaeza, Yaneth Castro-Coronel, Leonor C. Acosta-Saavedra, Luisa C. Hernández-Kelly, Daniel Hernández-Sotelo, Arturo Ortega

**Affiliations:** 1 Laboratorio de Neurotoxicología, Departamento de Toxicología, Centro de Investigación y de Estudios Avanzados del Instituto Politécnico Nacional, Av. IPN 2508, San Pedro Zacatenco, 07300 CDMX México; 2 Laboratorio de Epigenética del Cáncer, Facultad de Ciencias Químico Biológicas, Universidad Autónoma de Guerrero, Av. Lázaro Cárdenas 88, Chilpancingo, Guerrero, 39086 México

**Keywords:** RNA-binding proteins, Post-transcriptional modifications, Neurotoxicity, Neurodegenerative diseases

## Abstract

Despite sustained efforts to treat neurodegenerative diseases, little is known at the molecular level to understand and generate novel therapeutic approaches for these malignancies. Therefore, it is not surprising that neurogenerative diseases are among the leading causes of death in the aged population. Neurons require sophisticated cellular mechanisms to maintain proper protein homeostasis. These cells are generally sensitive to loss of gene expression control at the post-transcriptional level. Post-translational control responds to signals that can arise from intracellular processes or environmental factors that can be regulated through RNA-binding proteins. These proteins recognize RNA through one or more RNA-binding domains and form ribonucleoproteins that are critically involved in the regulation of post-transcriptional processes from splicing to the regulation of association of the translation machinery allowing a relatively rapid and precise modulation of the transcriptome. Neurotoxicity is the result of the biological, chemical, or physical interaction of agents with an adverse effect on the structure and function of the central nervous system. The disruption of the proper levels or function of RBPs in neurons and glial cells triggers neurotoxic events that are linked to neurodegenerative diseases such as spinal muscular atrophy (SMA), amyotrophic lateral sclerosis (ALS), fragile X syndrome (FXS), and frontotemporal dementia (FTD) among many others. The connection between RBPs and neurodegenerative diseases opens a new landscape for potentially novel therapeutic targets for the intervention of these neurodegenerative pathologies. In this contribution, a summary of the recent findings of the molecular mechanisms involved in the plausible role of RBPs in RNA processing in neurodegenerative disease is discussed.

## RNA-Binding Proteins: Overview

RNA-binding proteins (RBPs) can be defined as a wide group of proteins present in prokaryotic and eukaryotic cells that play a decisive role in regulating gene expression (Conti et al. 2017). Recently, RBPs have been described as the messenger ribonucleic acid (mRNA) clothes, as these proteins ensure that the different regions of mRNA (5′ and 3′ untranslated regions (UTR) and the coding region) could be either covered or exposed (Singh et al. 2015). These regulatory networks of RBP-mRNA-binding interactions, properly called ribonucleoprotein complexes (RNPs) (Thelen and Kye 2020), can remain stably bound throughout all post-transcriptional life of the mRNA (Lukong et al. 2008) and by these means allow numerous RNA interactions. Although these biochemical processes occur in numerous cells, these are especially essential in cells with complex RNA metabolisms, such as neurons and glial cells (Wolozin and Ivanov 2019).

The RNP association is extremely dynamic and prone to changes depending on the environment (Adeli 2011); any changes in some of the RNP could trigger cellular adaptive changes via modifications of the transcriptome and the proteome, meaning that RBPs are involved in the stabilization or decay of mRNAs in response to extracellular signals or stress (Alves 2016).

**Fig. 1:**
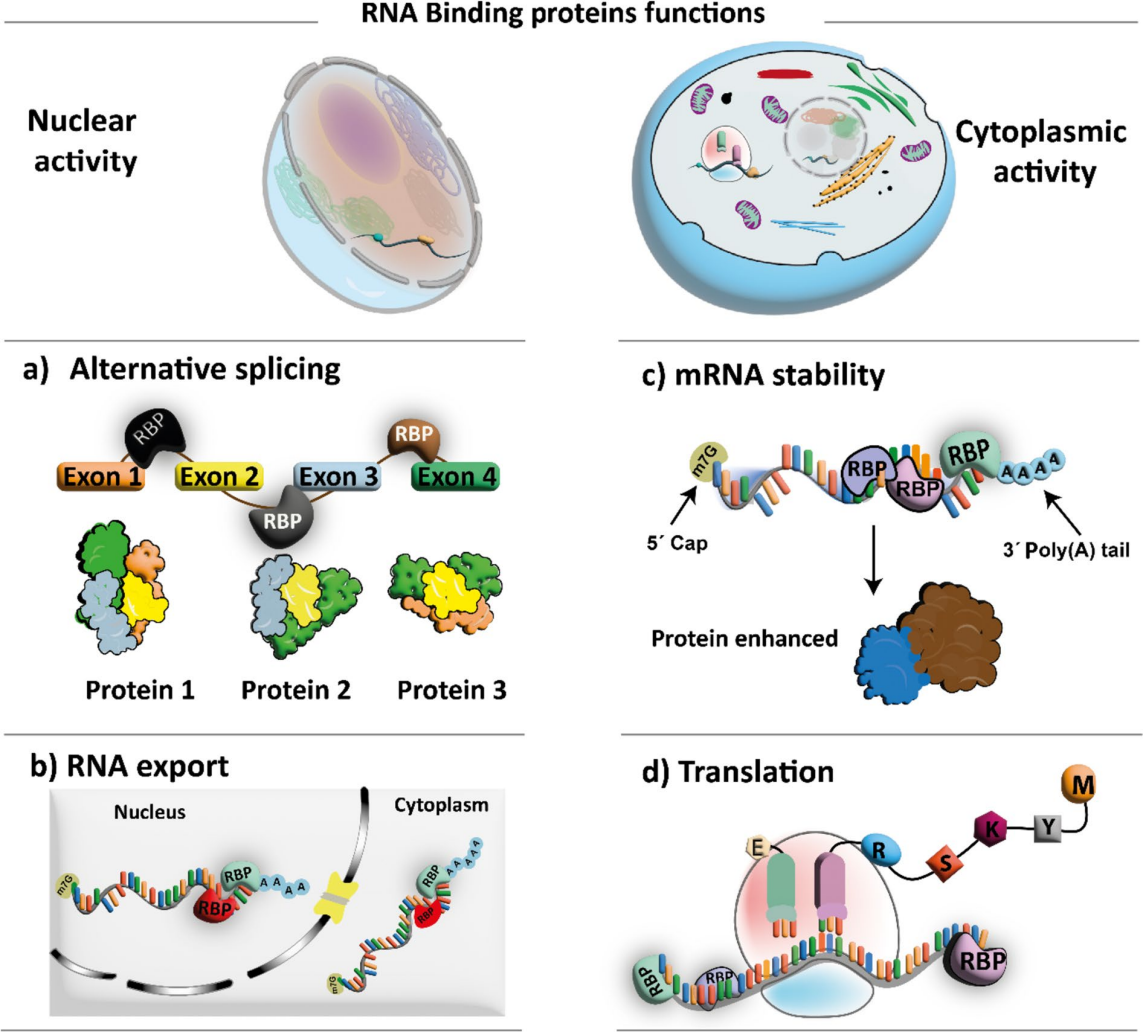
Representative summary of the different RBPs functions. The RBPs functions can be divided into nuclear and cytoplasmic activities. For example, in the nucleus, a) RBPs regulate the splicing of multi-exon genes and the exon skipping results in different protein isoforms from one unique gene. b) The RNA nuclear export by RBPs determines the proper out in the amount and correct timing from the nucleus. While in the cytoplasm, c) RBPs regulate mRNA stability and d) translation in the correct cytoplasmic localization

RBPs bind to the mRNA through a wide variety of intramolecular bonds in hairpins, stem loops, and other bumps and bulges (Attar 2014). Generally, RBPs associate with nascent mRNA both at the 5′- and 3′-ends. Their functions can generally be divided into nuclear and cytoplasmic activities. In the nucleus, RBPs regulate mRNA maturation, including RNA helicase activity, RNA polymerase elongation, splicing, and nuclear export. In the cytoplasm, RBPs regulate RNA transport, silencing, translation, and degradation (Halbeisen et al. 2008; Vanderweyde et al. 2013) (Fig. 1).

Despite the efforts to elucidate the number of RBPs expressed in eukaryotic cells, this number is still unknown; however, from studies based on bioinformatic strategies, it has been possible to calculate that between 2 and 8% of the number of total genes encode for RBPs (Gerstberger et al. 2014; Keene 2001).

It is important to note that not all RBPs share a common functional mechanism; it means that, while a subset of “housekeeping” RBPs could be constitutively and ubiquitously active, other subtypes are expressed in a more limited way such as those RBP involved in post-translational modifications (Xu et al. 2019), or being constantly inactive due to lack of their RNA targets (for example, due to the absence of RNA products from viral replication) (Garcia-Moreno et al. 2019).

Moreover, the regulatory roles of RBPs are also affected by the subcellular localization of RBPs and their RNA substrates (Nostrand et al. 2020). The transcripts are exported through nuclear pores to the cytoplasm in which RBPs may be targeted to specific subcellular regions by complexes consisting of motor proteins and RBPs or by a signal recognition particle (Halbeisen et al. 2008).

### Modular Structures of RBP

The plethora interactions of RBPs and mRNA result from a high degree of modularity, most of them contain more than one RNA-binding domain that is arranged in different modules to meet their diverse functional requirements (Lunde et al. 2007). Most RNA-binding proteins are built from few RNA-binding units and possess sequences of 2 to 6 nucleotides capable of binding to RNA-binding domains (RBDs) (Burd and Dreyfusst 1994). Multiple copies allow the recognition of larger and more complex RNA targets; in addition, these modules endow a protein with the ability to bind RNA with higher specificity and affinity in comparison with individual domains (Shotwell et al. 2020; Maris et al. 2005; Zhou et al. 2014).

### Functional Domains of RBP and their Properties

**Fig. 2:**
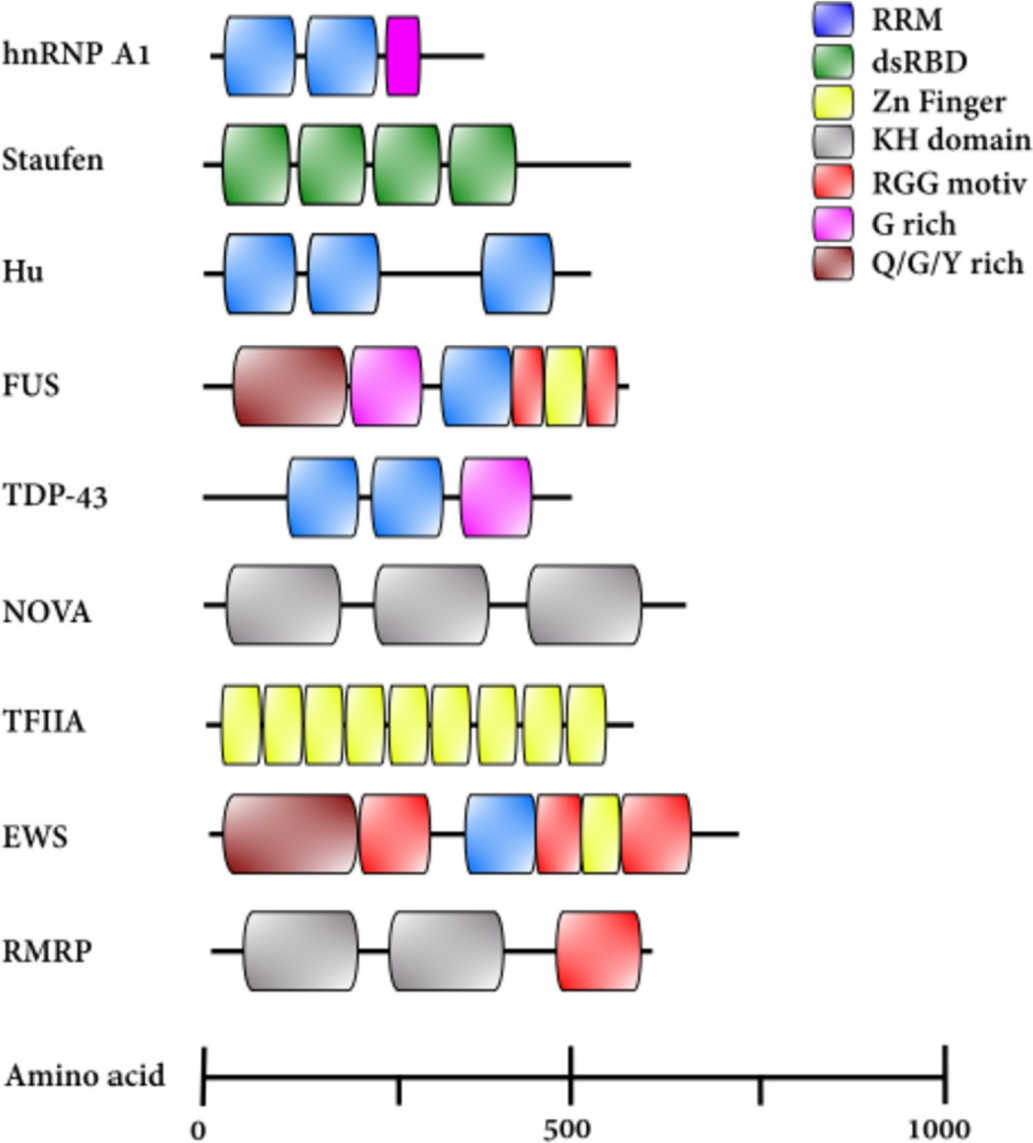
Modular structures of RBPs. Representative examples from some of the most common RNA-binding proteins involved in neurodegeneration. RNA-binding domains (RBDs) can act independently or when RBDs are found in multiple modules can act synergistically. Proteins are sized according to their amino acid lengths. RRM, RNA recognition motif; dsRBD, double-stranded RNA-binding motif; ZnF, zinc finger motif; KH, K-homology domain; RGG, Arg-Gly-Gly motiv; G, Gly motiv; Q/G/S/Y, Gln-Gly-Ser-Tyr motif. Modified from (Shotwell et al. 2020)

RBPs have one or more RNA-binding protein domains and share conserved domain structures and related functions (Vanderweyde et al. 2013); as shown in Fig. 2, most of these proteins fit the classical view of an RBP architecture with a modular combination of well-characterized RBDs and versatile RNA-binding surfaces (Beckmann et al. Jun 2016). The RBDs have been identified and classified according their substrate and structure: the RNA recognition motif (RRM), the zinc finger domain (ZnF), S1 domain, the K-homology domain (KH), the double-stranded RNA-binding domain (dsRBD), and Glycine-Arginine rich (RGG) (Conti et al. 2017; Chen and Varani 2005; Glisovic et al. 2008).

#### RNA Recognition Motif Domain

The RNA Recognition Motif (RRM) domain is an abundant domain and the most studied both in terms of structure and biochemistry. Genome sequencing studies have provided evidence that RRM-containing proteins are present in all forms of life (Maris et al. 2005; Afroz et al. 2015; Oliveira et al. 2017). To date, more than 10,000 RRMs have been identified that function practically in all post-transcriptional gene expression processes; in humans, ~ 0.5–1% of genes contain an RRM, often in multiple copies of the same polypeptide (Kuo et al. 2014).

Modified from Shotwell et al. (2020) The RRM is composed of a stretch of 80–90 amino acids that form a four-stranded antiparallel β sheet with two helices forming a divided αβ (βαββαβ) topology (Oubridge et al. 1994). The binding is mediated in most cases by an arginine (R) or a lysine (K) residue that forms a salt bridge to the phosphodiester backbone and two aromatic residues that make stacking interactions with nucleobases (Chen and Varani 2005). A single RRM can recognize a short sequence of nucleotides (between four and eight) due to the presence of exposed loops (Varani and Nagai 1998). Although some RNAs can bind to individual RRMs with high specificity, multiple domains are often required to define specificity because the number of nucleotides that are recognized by a single RRM is generally too small to define a single binding sequence (Duszczyk et al. 2019; Jankowsky and Harris 2015).

#### Other Binding Domains

The KH domain is approximately 70 amino acids long and binds four nucleotides. Two versions of the KH fold have been reported, type I and type II, which are found in eukaryotic and prokaryotic proteins, respectively (Valverde et al. 2008). The dsRBMs were first described as recognizing an RNA shape rather than an RNA sequence, and these domains contain approximately 70 amino acids and exhibit a conserved αβββα protein topology. These domains all interact along one face of a regular α-helix structure and can cover up to 16 bp spanning two consecutive minor grooves separated by a major groove (Chang and Ramos 2005; Stefl et al. 2005).

A classical ZnF is about 30 amino acids long and displays a ββα protein fold in which a β-hairpin and an α-helix are pinned together by a Zn^2+^ ion*.* In a single RBP, this motif can be found alone as a repeated domain or it can interact specifically with dsDNA motifs bases located in major grooves via side chains of residues present in their α-helix (Wolfe et al. 2000).

Low-complexity regions (LCR) are enriched mainly by serine (S), proline (P), glycine (G), arginine (R), lysine (K), and tyrosine (Y) (Toll-Riera et al. 2012). These amino acids form definite patterns: G often coexists with R or Y, generating repeats of RG or YG that can appear multiple times within a given protein region resulting in highly repetitive sequences. Both S and P have a high propensity to be in disordered regions (Schwartz et al. 2021). Many of the low-complexity regions in RBDs have been predicted to be intrinsically disordered regions (IDRs) that natively lack a three-dimensional stable structure (Calabretta and Richard 2015; Habchi et al. 2014; Uversky 2019).

## RNA Processing and Regulation by RBPs

### Crucial Role of RBP in mRNA Stability

After mRNA transcription in the nucleus, RBPs recognize pre-mRNA to regulate alternative splicing, polyadenylation, or stability. RPBs play an important role in mRNA stability; to make this possible, the RRM binds selectively to elements rich in adenylate and uridylate (ARE) in the 3′UTR region of mRNAs (Glisovic et al. 2008; Sena et al. 2021). AREs are present in 5–8% of human genes with various functions such as cell growth and differentiation, signal transduction, apoptosis, nutrient transport, and metabolism (García-Mauriño et al. 2017).

When the mature mRNA is transported to the cytoplasm, both its stability and distribution to different cell compartments can be modified by the different interactions with RBPs (Matoulkova et al. 2012). The final fate of the mRNA depends on the signaling pathway associated with the binding of the RBP in question; in general, RBPs can modify the half-life of the mRNA, that is, they can stabilize or destabilize it (Matoulkova et al. 2012). For example, proteins such as heterogeneous nuclear ribonucleoprotein D0 (hnRNPD0), tristetraprolin (TTP), TIA-1 T-cell internal antigen-1 (TIA-1), TIA-1 related protein (TIAR), and K-homology splicing regulatory protein (KSRP) bind to AREs and destabilize the mRNA, while the different proteins of the Hu family such as human antigen R (HuR) stabilize the mRNA as they delay the initiation of disintegration (García-Mauriño et al. 2017). Furthermore, with the help of RRM and the nuclear transport sequence (HNS), HuR binds to the target mRNAs in the nucleus, exports and protects it during cytoplasmic transit, and facilitates its recruitment into the translation machinery. This leads translation initiation increase the the mRNA stability (Fan and Steitz 1998). Interestingly, HuR proteins are susceptible to post-translational modifications. Their phosphorylation by protein kinase C (PKC) leads to increased translocation to the cytoplasm, resulting in altered cellular processes (Grammatikakis et al. 2017). Therefore, the presence of a nucleus or cytoplasmic HuR determines the normal or pathological state of a cell in the context of various physiological and pathological stimuli (Suresh et al. 2016).

### RBPs, 3′UTRs, and poly (A) Tail of mRNA

Polyadenylation is an exquisite process that consists in the addition of a poly (A) tail to the mRNA by poly (A) polymerase; this phenomenon generates an effect on its nuclear transport, translation efficiency, and stability. All eukaryotic mRNAs, except the replication-dependent histone mRNAs, are polyadenylated at their 3′ ends in a process associated with transcriptional termination. It has been demonstrated in eggs and early embryos that mRNAS with longer poly (A) tails are more efficiently translated (Bartel and Xiang 2021; Moore and Lindern 2018). The binding of poly A-binding proteins (PABP) to the poly (A) tail is critical for the translation initiation. PABPs interact directly with the eukaryotic translation initiation factor 4G (eIF4G) scaffold protein of the eukaryotic initiation factor 4F (eIF4F) cap-binding complex (Brook and Gray 2012; Hinnebusch and Lorsch 2012). This brings the mRNA tail closer to the cap and forms an mRNA loop that optimizes the recycling of translation initiation and elongation factors (Neelagandan et al. 2020; Sonenberg and Hinnebusch 2009). Furthermore, the binding of PABP to the poly (A) tail protects from degradation, and the length of the poly (A) tail also affects the initiation of translation (Norbury 2013; Rissland et al. 2017).

The cytoplasmic polyadenylation element-binding protein (CPEB) is an RBP present in the cytoplasm responsible for the recruitment of poly (A) polymerase that has two RRM motifs and two ZnF (Hake et al. 1998; Ivshina et al. 2014; Kozlov et al. 2021), and by binding to the cytoplasmic polyadenylation element (CPE) 3′UTR, it recruits a series of proteins that interact to modulate the length of the tail of poly (A) resulting in positive regulation of translation (Richter 2007; Szostak and Gebauer 2013).

During development and in the adult organism, RBPs have key roles in the polyadenylation process. Alternative cleavage and polyadenylation (APA) lead to the expression of different isoforms of a same gene that might play a role in the etiology of a particular disease (Xing et al. Mar 2021). There are differential 3′UTRs lengths between cells in the organism that participates in the polyadenylation and cleavage regulation, for example, neurons are known to have longer 3′UTRs, while microglia and endothelial cells express shorter UTRs (Guvenek and Tian Sep 2018). The differential binding of RBPs at 3′UTR regulates the recruitment or prevents polyadenylation. Neuro-oncological ventral antigen (Nova) is another RBP that was found to bind 3′UTRs and enriched near poly (A) sites in the mouse brain. The comparison between *Nova2* wild-type and knockout results in alternate 3′ UTR changes relative to total mRNA abundance, due to overlap with the canonical cleavage and polyadenylation specificity factor (CPSF) and/or cleavage stimulatory factor (CstF) binding sites of the *Cugbp2* and *Slc8a1* poly(A) sites, which are suppressed by Nova (Licatalosi et al. 2008). Muscleblind like splicing regulator 1 (MBNL) is another RBP essential polyadenylation regulator in mouse embryo fibroblasts; its depletion leads to dysregulation of thousands of alternative polyadenylation events (Batra et al. 2014).

RNA-binding protein fused in sarcoma (FUS) is frequently found around alternative polyadenylation (APA) sites of nascent RNA. APA sites located upstream of FUS cluster enhances polyadenylation by recruiting CPSF160 and up-regulates the alternative short transcript. In contrast, APA sites located downstream from FUS cluster polyadenylation is not activated; RNAP II-suppressing effect of FUS leads to down-regulation of the alternative short transcript. The regulation of mRNA lengths by FUS is operational in two-thirds of transcripts in neuronal cells, with enrichment in genes involved in synaptic activities (Masuda et al. 2015). In a structural and *cis* sense, the 3′UTR are key regions pre-mRNA regulated by RBPs for polyadenylation and splicing events.

### RBP Association with Splicing Regulation

RBPs can regulate gene expression from the splice site through their binding to RNA (Yee et al. 2019). Constitutive splicing is the process of intron (non-coding segments) removal and exon (coding segments) ligation in the order in which they appear in a gene. Alternative splicing is a deviation from this preferred sequence resulting in various forms of mature mRNA (Wang et al. 2015). Most human intron deletions are catalyzed by a large RNP complex called a spliceosome. It is estimated that 95% of human genes are alternately spliced (Fredericks et al. 2015; Lee et al. 2015).

The two major RBPs splicing factors are the heterogeneous ribonucleoprotein particles (hnRNPs) and serine-arginine (SR) proteins (Fredericks et al. 2015). Although hnRNPs and SR proteins are believed to be the main splicing factors that regulate RBP, RNA-binding protein fused in sarcoma (FUS) has recently been implicated (Humphrey et al. 2020). Although there remains a class of uncharacterized hnRNP proteins, over 50% have been characterized to play a role in splicing (Han et al. 2013). SR proteins are distinguished due to their domain found near the C-terminal domain that promotes protein–protein interactions between the SR protein and the spliceosome (Zheng et al. 2020). Many RBPs regulate the skipping or inclusion exon according to downstream or upstream binding site, for example Nova 1 and 2, RNA-binding protein fox-1 (FOX-1), and polypyrimidine tract-binding protein (Ptbp) 1 and 2 (Ule et al. 2006; Raj and Blencowe 2015). Any change in the *cis* sequence and levels of trans-factors can alter splicing and cause disease.

### Mechanisms of RBP-related Splicing Dysregulation

#### Pre-mRNA Mis-splicing

Removal of introns from pre-mRNAs is a *sine-qua-non* process for the expression of most human genes. Pre-mRNA splicing and its regulation require a complex array of *cis-elements* (the splicing code) embedded in pre-mRNAs and *trans* factors that bind to these elements (Chao et al. 2021).

Alternative splicing is influenced by interactions between RNA sequences and the surrounding sequence contexts: exonic splicing enhancer (ESE), exonic splicing silencer (ESS), intronic splicing enhancer (ISE), and intronic splicing silencer (ISS) elements and their binding *trans­regulatory* factors (e.g., splicing factors and RBPs) (Chao et al. 2021; Singh and Cooper 2012). RNA recognition motifs interact with single-stranded RNA targets using their β-sheet surface. Diseases caused by point mutations can alter splicing by the disruption of *cis-elements* that modulate the recognition of the splice sites. These auxiliary elements are often ligands for RBPs (Fredericks et al. 2015). The main RBPs involved in alternative splicing are SR and hnRNP. SR proteins bound in the exon are generally regarded as activating splicing whereas the same protein at the intron can act as a repressor. Conversely, hnRNPs are regarded as repressors when bound to exonic locations and activators when bound to the intron (Erkelenz et al. 2013). ESE motifs functionally repress splicing when are found in the intron, becoming intronic splicing silencers (Martinez-Contreras et al. 2006). Likewise, ESS motifs have been shown to function as intronic splicing enhancers (Kanopka et al. 1996). Mutations that increase the stability of interactions between an RNA species and RBP substrate can cause disease. This has been demonstrated in several *well-studied* diseases particularly neurological and muscular degenerative disorders. Often, the repeated sequence becomes pathogenic after expanding beyond a threshold length. The toxic mRNA transcripts produced cause the dysregulation of alternative splicing of many pre-mRNAs in *trans* simultaneously, also known as *spliceopathy* (Afroz et al. 2015; Boo and Kim 2020).

#### Splicing Factors Alteration

Another category of *spliceopathy* is the direct mutation of a splicing factor. NOVA and TAR DNA-binding protein 43 (TDP-43) are the two RBPs in this category of *spliceopathy*, regulate alternative events in neurons, and the loss of either of them results in severe pathogenesis (Boo and Kim 2020; Kapeli et al. 2017). Loss of Nova proteins, as a result of an autoimmune paraneoplastic neurological disorder, manifests itself in neurological symptoms of excess motor movements (paraneoplastic myoclonic opsoclonus ataxia, POMA), on other hand, TPD-43 has been involved in ALS pathology (Buckanovich et al. 1993).

## RBP are Involved in Stress Granules

In eukaryotic cells, stress conditions such as accumulation of reactive oxygen species (ROS) often inhibit the initiation of translation and trigger the formation of cytoplasmic RNA–protein complexes called stress granules (SG) (Markmiller et al. 2018) that store RNA and that is released when physiological conditions are restored (Shashidharan et al. 1997; Schieweck et al. 2021). These granules are present in the pathophysiology of neurodegenerative diseases, particularly amyotrophic lateral sclerosis, frontotemporal dementia, and Alzheimer’s disease (AD). SGs contain 40S ribosomal subunits, mRNA, translation initiation factors, and RBPs (Wolozin and Ivanov 2019; Markmiller et al. 2018) so that post-translational modifications of RBP can directly modify the regulation of SGs (Anderson and Kedersha 2009). Recent studies show that few nuclear RBPs are expressed under neuropathological conditions, and a higher fraction is present in SG (Apicco et al. 2018; Armstrong et al. 2012; Janssens et al. 2013). Through proteomics, it has been demonstrated that the following factors contribute to the assembly of SG:

Interaction with the G3BP stress granule assembly factor 1 (G3BP1) protein: RNAs containing G-quadruplex (GGGGCC) act as molecular scaffolds to recruit specific RBPs such as G3BP1, increasing their local concentrations and promoting SG (Hofmann et al. 2020; Fay et al. 2017; Jain et al. 2016).

**Fig. 3:**
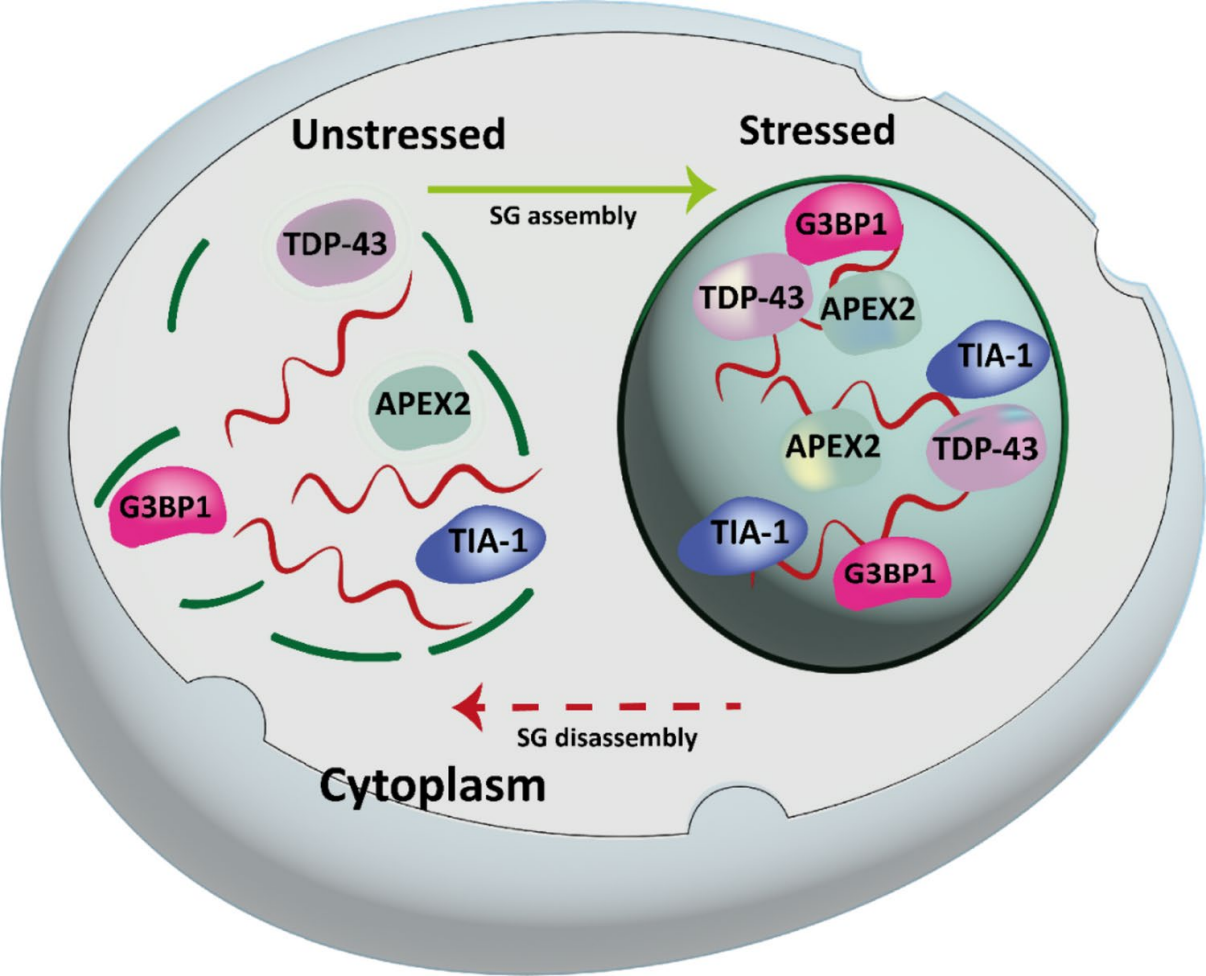
Assembly and disassembly of stress granules. Under unstressed conditions, mRNA exists in the cytoplasm and is normally translated. Upon stress, mRNAs are protected within stress granules. Once the stress has been removed, the stress granules disassemble. The dynamic assembly of SG is also promoted by RBPs such as TIA-1. TDP-43, TAR DNA-binding protein 43; TIA-1, T-cell internal antigen-1; G3BP1, stress granule assembly factor 1; APEX2, apurinic/apyrimidinic endodeoxyribonuclease 2. Modified from (Hofmann et al. 2020)

The dynamic assembly of SG is also promoted by RBPs such as TIA-1 and TIAR since these proteins are capable of dimerizing and promoting polysome assembly (Fig. 3) (Martin and Tazi 2014) in response to phosphorylation of the eukaryotic initiation factor 2 alpha (eIF-2α). TIA-1 and TIAR have 3 RRMs which confer the necessary functions for the assembly of SG (Kedersha et al. 2000; Panas et al. 2016).

SG assembly can also be affected by changes in levels of ubiquitination (Cao et al. 2020) or methylation (Xie and Denman 2011). Stress granules also interact with P-bodies (PB), another dynamic cytoplasmic RNA protein structure found in the cytoplasm of eukaryotes. PB contains components of the mRNA decomposition machinery (Buchan and Parker 2009).

**Fig. 4:**
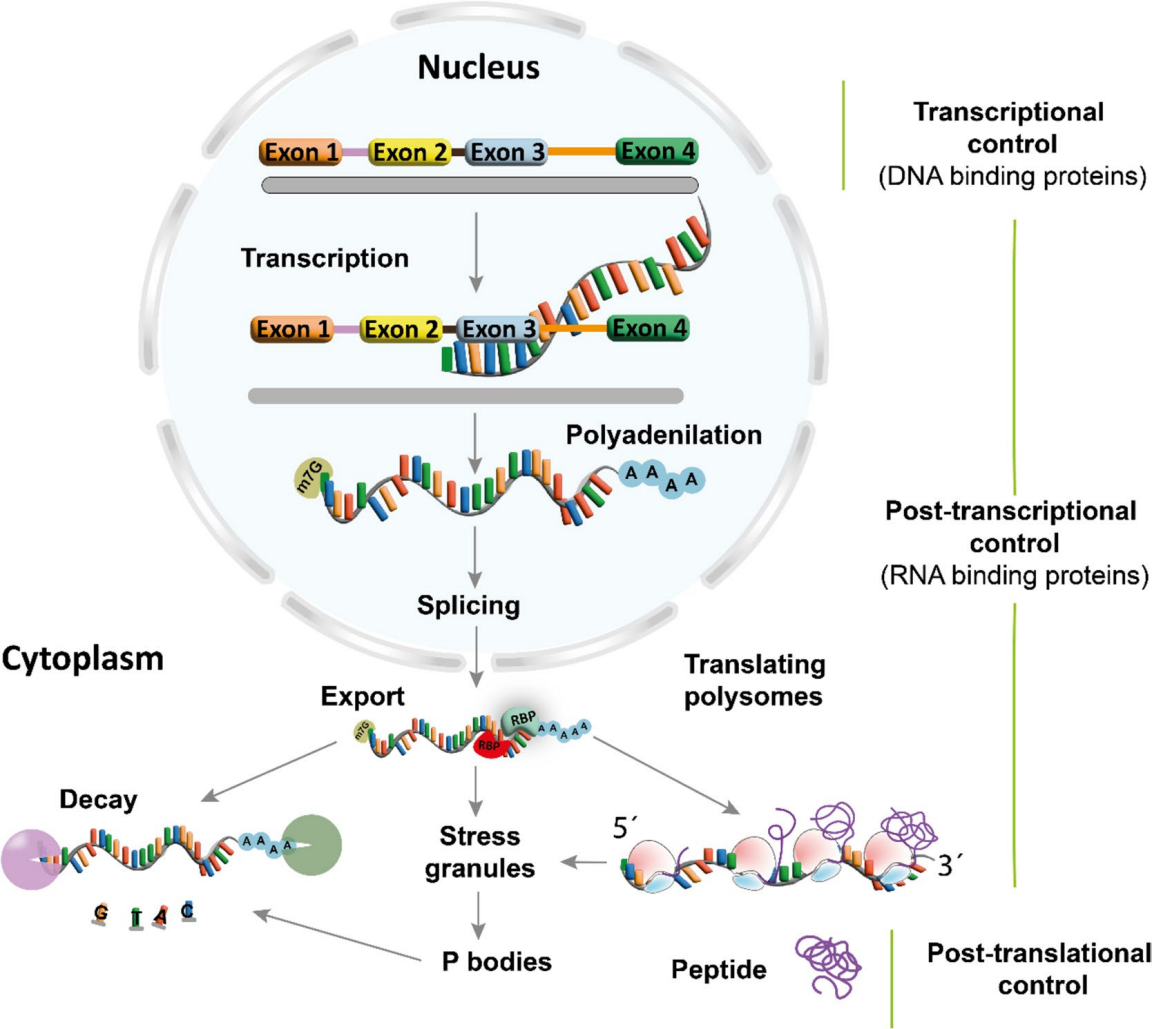
RNA regulation cascade by RBPs from the nucleus to the cytoplasm. From DNA to RNA transcription, there are RBPs involved in the isoform length by two mechanisms: the alternative exons selection (splicing) and the alternative polyadenylation sites. After RNA export at the post-translational level is regulated by some RBPs that can conduce to decoy, protection mechanisms such as the formation of dynamic stress granules and P-bodies or ensure the performance of mRNA through translating polysomes that ensure a high peptide-protein expression rate, all processes in at post-translational level

SG and PB interact physically, suggesting possible traffic of stored mRNA between these compartments (Parker and Sheth 2007). PB recruits mRNA for translation control (Hubstenberger et al. 2017) and degradation (Fig. 4) (Decker and Parker 2012).

### SG Disassembly

SGs contain non-translatable mRNAs that are classified and processed according to metabolic status and environmental changes, and these mRNAs can be activated when stressful conditions dissipate repression or decomposition. Theoretically, SG could be disassembled by dissociating the interactions by transferring the material to a PB or removing the mRNAs from SGs by entering the polysomes (Markmiller et al. 2018; Anderson et al. 2015).

## RBP are Essential for Brain Function

To ensure a proper neuronal development and synaptic plasticity, the brain contains the highest amount of transcriptional and post-transcriptional mechanisms described thus far (Pilaz and Silver 2015). Following RNA splicing and the first step of quality control in the nucleus, mRNAs will be exported through the nuclear pore into the cytoplasm, where the interaction with different RBPs ensures proper control of localization, stability, and translation (Darnell and Richter 2012; Soheilypour and Mofrad 2018). The neuronal transcriptome is enormously diverse due to alternative splicing, polyadenylation, intron retention, and the occurrence of non-canonical coding sequences (Sibley et al. 2016). To guide the expression of the transcriptome, multiple RBPs will dynamically interact in a spatially and temporally defined as well as cell type-specific manner which explains the great variety of RBPs in cells (Schieweck et al. 2021). RBPs bound to mRNAs and are associated with motor proteins to specific localizations in *membrane-less* and *shape high* molecular weight complexes (mRNP). The kind of RBP determines the subcellular location of their components including mRNAs. In the brain, the axonal cone growth has a differential translational where the RBPs CPEB1 (Richter 2007) and ZBP1 (Huttelmaier et al. 2005) are critical mediators of mRNA transport and its translation. ZBP1 assembly β-actin mRNA to direct localization in axons and dendrites (Song et al. 2015). *Staufen* is another RBP involved with RNA granules moving along microtubules into dendrites of hippocampal neurons in a bidirectional manner (Kohrmann et al. 1999). The fragile X mental retardation protein (FMRP) interacts with kinesin to dendritic mRNA localization and regulates the local translation in these sites (Wang et al. 2016; Dictenberg et al. 2008). Pumilio 2 (Pum2) acts like FMRP as a translational regulator, and its specific localization is related to repressor function by inhibiting translation and promoting mRNA decay (Goldstrohm et al. 2018).

### RBPs Dysregulation: Triggering the Disease

From the progress in genetic studies, it has been established that RBP dysregulation or mutation can trigger loss of neurons, neuronal function, and neurodegeneration (Kapeli et al. 2017). Nowadays, more and more RBPs are being recognized as causal factors or associated with neurological diseases, autoimmunity, and cancer (Wolozin and Ivanov 2019; Van Nostrand et al. 2020) reinforcing the importance of RBPs in the maintenance of the normal physiology of the CNS. A considerable amount of RBPs have LCR, so these proteins are prone to structural modifications and consequently trigger the loss or gain of function, contributing to the severity of neurodegeneration (Kapeli et al. 2017). Despite the complexities that RBPs currently represent, the binding sites in RNAs could provide more detailed information on the development of neuronal diseases that involve RBPs, which would undoubtedly be an important contribution to this public health problem (Pan et al. 2020).

Mutations in genes encoding RBP have been observed in patients with motor neuron disorders such as amyotrophic lateral sclerosis (ALS), spinal muscular atrophy (SMA), multisystem proteinopathy (MSP), and frontotemporal degeneration (FTD); of all these, ALS is the most common motor neuron disorder in adults; this condition is characterized by the progressive loss of motor neurons triggering fatal paralysis (Robberecht and Eykens 2015).

In neurons, mRNAs can be transported to and from axons and dendrites as mRNA can be translated locally. In neuronal processes, such as dendrites, there are a great variety of mRNAs and a large part of the transcriptome is present both in dendrites and in axons. In the vertebrate brain, mRNAs containing localization elements or *“zip codes”* have been identified in neuronal processes, including those encoding structural proteins (Tiruchinapalli et al. 2003), receptors (Grooms et al. 2006), and signaling molecules (Martin and Ephrussi 2009; Terenzio et al. 1421). These *cis*-acting sequences located in the 3′UTR are generally recognized by RBPs and will assemble into an RNP (Turner-Bridger et al. 2020). The *cis* information contained in the 3′UTR mRNAs is essential to the RBPs recognition; the activated leukocyte cell adhesion molecule (ALCAM) mediates homophilic adhesion of axons from the same neuronal subtype and is required for the formation of axon bundles. The lack of its 3′UTR results in overexpression and induces axon bundle aggregation and prevents axonal growth, whereas a decreased expression results in fasciculation (Thelen et al. 2012), accordingly. The full length of ALCAM maintains the right amount.

The RNP complex can mediate interactions with the translation machinery and self-assemble into transport granules (Eliscovich and Singer 2017). One of the most abundant RNPs in both axons and dendrites is β-actin, and its association with the *zip code* binding protein 1 (ZBP1) is essential for transport and proper localization (Biswas et al. 2019; Das and Yoon 2019).

Late endosomes serve as a platform for local axonal translation by binding to RBP, ribosomes, and mRNA (Cioni et al. 2019). Another interesting cellular component that regulates neuronal protein synthesis are RNA granules. Neurons contain various RNA granules, including SG and PB (Thelen and Kye 2020). Importantly, it has been shown that RNP granules located in dendrites can disassemble and mRNAs can be used as a template to produce synaptic proteins (Schieweck et al. 2021; Krichevsky and Kosik 2001). This process is crucial for neuronal health and function, as it is a cellular homeostatic mechanism for managing external stress and controlling synaptic plasticity. These mechanisms will directly impact the composition of the local proteome (Das and Yoon 2019; Jung et al. 2014).

As RBPs determine the axonal or dendritic mRNA *repertoire* as well as proteomes by trafficking mRNAs and regulating local protein synthesis, RBP plays a crucial role in neuronal function. Dysfunctional RNA processing in neuronal tissue plays a crucial role in neuronal pathology and is often observed in neurodegenerative diseases (Thelen and Kye 2020). Moreover, RNP plays an important role in RNA metabolism, regulating ribosome formation, spliceosomes, and silencing complexes. When gene mutations or deletions occur in neurons or a well-*misregulated* assembly of RNP occurs, it results in neuron degeneration which can lead to SMA, ALS, fragile X syndrome (FXS), among other pathologies (Shukla and Parker 2016).

Due to complex neuronal compartmentalization, it is indispensable to provide local mRNA transcripts and make neurons vulnerable to any change and loss of RNPs complex. Neurons can synthesize proteins at the synaptic compartment in response to many stimuli. For example, the cellular components necessary to produce proteins such as ribosomes and mRNAs are detected at the synaptic area (Ainsley et al. 2014; Poulopoulos et al. 2019). Thus, RNPs deliver specific sets of mRNAs and produce different proteins in particular subcellular compartments, due to the motifs that provide an accurate function of RBP and RNPs at local mRNA translation. These processes are crucial for neuronal development and function (Jung et al. 2014).

### Role of RBPs in Neurotoxicity

The brain is susceptible to damage by several toxic agents such as metals, microorganisms, persistent organic pollutants, and high levels of glutamate. RBPs as key regulators of transcriptome are found deregulated in many neurotoxic diseases. In FTD and ALS, there are abnormal controls of mRNA translation by TDP-43 and FUS accumulation in SGs. The motor neuron degeneration in these diseases is related to mutations in RBPs genes (Gowell et al. 2021; Zhou et al. 2020; Vance et al. 2009).

The abnormal cytoplasmic accumulation of TDP-43, correctly called TDP-43 proteinopathy, contributes to neurotoxicity and the oligonucleotides treatment composed of TDP-43 target sequences rescues neurotoxicity (Schieweck et al. 2021; Mann et al. 2019). ALS-associated mutations in TDP-43 are frequently found in LCR Gly-rich domains that regulate phosphorylation and ubiquitination sites (Pesiridis et al. 2009). The prion-related domains rich in glutamine(Q) and asparagine (N) present in TDP-43, TIA-1, and FUS are associated with a highly prone to aggregation (Udan and Baloh 2011). As described above, the cytosolic accumulation of almost any RBPs and the disruption of their nuclear functions is a triggering feature of neurotoxicity. For example, wild-type human TDP-43 can be toxic when expressed in a heterologous *C. elegans* system or overexpressed in a cell culture model (Ash et al. 2010). In mice, TDP-43 mutant alleles cause dose-dependent asymmetrical motor axon withdrawal and the lethality and cognitive dysfunction are rescued with functional TDP-43 (Ebstein et al. 2019). The wild-type human TDP-43 expression causes mitochondrial aggregation, motor deficits, and early mortality in transgenic mice (Xu et al. 2010). In chick embryo models, TDP-43_Q331K_ and TDP-43_M337V_ showed a dramatic reduction in maturation compared to TDP-43WT with a failure to develop normal limbs and tail buds (Sreedharan et al. 2008). The lacking TDP-43 in flies results in deficient locomotive behaviors, life span reduction, and anatomical defects at the neuromuscular junctions (Feiguin et al. 2009). Some studies report that TDP-43 mutations are more neurotoxic compared to wild-type TDP-43; however, it is necessary to emphasize that a mutation in this RBP is not necessary to promote ALS (Gregory et al. 2020; Wegorzewska et al. 2009). Although some studies report neurodegeneration in the absence of cytosolic aggregation how consequence from TDP-43 specific localization to motor neuron nuclei (Hanson et al. 2010).

From RNA interference screening, the inositol-1,4,5-triphosphate receptor type 1 (ITPR1, mediator of Ca^2+^ efflux) was identified as a new regulator of nucleocytoplasmic transport of TDP-43 since the silencing of this receptor promotes the cytosolic accumulation of TDP-43. Therefore, these findings also suggest that the expression and localization of TDP-43 are regulated by Ca^2+^ (Kim et al. 2012). Duan et al. (2019) revealed that PARylation levels are an important regulator of assembly and disassembly dynamics of RNP granules containing hnRNP A1 and TDP-43. They also showed that both genetic and pharmacological inhibition of PARP mitigates neurotoxicity mediated by hnRNP A1 and TDP-43 in cellular and Drosophila models of ALS. At the same time, PAR binding through the hnRNP A1 PAR-binding motif regulates its association with stress granules (Duan et al. 2019).

Mutations in Matrin 3 (MATR3), a DNA and RNA-binding protein little studied so far, have also been described as causing ALS and FTD. Using a primary neuron model to evaluate MATR3-mediated neurotoxicity, Malik et al. (2018) showed that neurons were bidirectionally vulnerable to MATR3 levels. In addition, the ZnF MATR3 domains partially modulated toxicity; however, the elimination of their motifs for RNA recognition did not affect neuronal survival. On the other hand, contrary to other RBPs related to ALS, the cytoplasmic redistribution of MATR3 mitigated neurodegeneration, suggesting that nuclear MATR3 mediates toxicity (Malik et al. 2018).

Another of the main cause of ALS and FTD is the expanded GGGGCC (G_4_C_2_)_n_ repeats in the first intron of the *C9orf72* gene; this repetition promotes a gain of function that undoubtedly alters the homeostasis of post-transcriptional processes. Celona et al. (2017) identified Zfp106, a ZnF domain RBP, as a specific 4G RNA repeat binding protein. Furthermore, the authors showed that Zfp106 interacts with other RBPs. Zfp106 potently suppresses neurotoxicity in a *Drosophila* model of ALS C9orf72 (Celona et al. 2017). Another RBP in these diseases is the RNA editing enzyme adenosine deaminase acting on RNA 2 (ADAR2), which is mislocalized in C9orf72 repeat expansion = mediated ALS/FTD. Because of this mislocalization, severe RNA editing alterations were observed in multiple brain regions. The mislocalization of ADAR2 in C9orf72-mediated ALS/FTD is responsible for the alteration of RNA processing events that may impact vast cellular functions, including the integrated stress response (ISR) and protein translation (Moore et al. 2019).

In autism disorder (ASD), CPEB4 regulates the translation of specific mRNAs by modulating their poly(A)-tails, and it was found to bind transcripts of most high-confidence ASD risk genes. Individuals with idiopathic ASD show imbalances in CPEB4 transcript isoforms, and 9% of the transcriptome shows reduced poly(A)-tail length (Parras et al. 2018). In the same disease, functional defects of the cerebral cortex contribute to the clinical symptoms of ASD, and impairment of Rbfox1-iso1 is a main effector. The Rbfox1-iso1 knockdown in hippocampal neurons resulted in the reduction of primary axon length, total length of dendrites, spine density, and mature spine number with an important impact on neuronal migration and synapse network formation during corticogenesis (Hamada et al. 2016).

Taken together, the literature supports that RBPs are key regulators in many neurotoxicology diseases; in Table 1, we summarized the association of different RBPs dysfunction and process altered in neurological disorders.

**Table 1:** RBP dysfunction and process altered in neurological disorders

RBP/RBD	Neurological disease	Biological mechanisms	Ref
FUS: RRM, G rich, Q/G/S/Y, ZnF, RGG	ALS FTD	Alternative splicing Transport	Gowell et al. 2021; Zhou et al. 2020; Vance et al. 2009)
Rbfox: RRM	Epilepsy, ASD, and mental retardation	Alternative splicing Polyadenylation	Hamada et al. 2016; Rajman et al. 2017; Lee et al. 2016; Jin et al. 2003)
PABP: RRM	MD	Alternative splicing Stability mRNA	Banerjee et al. 2013; Schoser and Timchenko 2010)
HuR/ELAVL1: RRM	SSN, diabetic nephropathy, glioma progression and PEM	Stability, alternative splicing, polyadenylation, 3′UTR binding and transport	Zhu et al. 2007; Filippova et al. 2017; Ince-dunn et al. 2012)
U1A: RRM	SMA	Inhibits polyadenylation upon direct binding to mRNA	Workman et al. 2014)
TDP-43: RRMG rich	ALS and FTD	Alternative splicing, miRNA biogenesis, stability, and transport	Jo et al. 2020; Deshaies et al. 2018; Neumann et al. 2006)
CPEB: RRM	ASD	Polyadenylation	Parras et al. 2018)
TIA-1: RRM/KH	ALS	Alternative splicing Apoptosis promotor via FAST-K	Wang et al. 2014; Rayman and Kandel 2017)
ZBP1: RRM/KH	Guide, growth and branched axon, dendritic development, synaptogenesis, and regeneration	3′UTR binding and stability, translational repression Axonal mRNA transport, localization, and degradation	Gallagher and Ramos 2018; Bryant and Yazdani 2016)
Nova: KH	FXS	Alternative splicing Polyadenylation	Yang et al. 1998; Park et al. 2011; Lewis et al. 2000)
FMRP: KH/RGG	FXS	Alternative splicing, mRNA stability, dendritic mRNA transport, and local postsynaptic protein synthesis	Burd and Dreyfusst 1994; Yang et al. 2018; Hall and Berry-Kravis 2018; Telias 2019)
hnRNP: KH RGG	ALS, FTD, Kabulki syndrome, and Au-Kline syndrome	Transcription, silencing 3′UTR binding and stability	Wang et al. 2019; Geuens et al. 2016; Bampton et al. 2020)
QK1: KH	Schizophrenia and ataxia	Stability, translation, alternative splicing, and localization	Hayakawa-Yano et al. 2017; Lauriat et al. 2008; Hardy 1998)
STAU1: dsRBD	MD and AD	Alternative splicing and 3′UTR binding	Zhong et al. 2020; Yu et al. 2015; Bondy-Chorney et al. 2016)
Adar 1/2: dsRBD	ALS, FTD, and IPF	miRNA processing and alternative splicing	Moore et al. 2019; Bryant and Yazdani 2016; Barraud and Allain 2012)
EWS: RGG	ALS, FTD, and Ewing sarcoma	Alternative splicing	Shaw et al. 2010; Selvanathan et al. 2015)
ATX2: RRM	ALS, SCA2, ELA, and FTD	Polyadenylation mRNA stability SG and PB formation	Zhou et al. 2014; Ostrowski et al. 2017; Watanabe et al. 2020; Nonhoff et al. 2007)

*AD* Alzheimer disease, *ALS* amyotrophic lateral sclerosis, *ASD* autism spectrum disorder, *COPD* chronic obstructive pulmonary disease, *FTD* frontotemporal lobar dementia, *MD *myotonic dystrophy, *PB* P-bodies, *POMA* spinal muscular atrophy, *SCA2* spinocerebral ataxia type 2, *IPF* idiopathic pulmonary fibrosis, *SSN* subacute sensory neuropathy, *PEM* paraneoplastic encephalomyelitis, *FXS* fragile X syndrome, *SG* stress granules, *PB* P-bodies, *SMA* spinal muscular atrophy, *RRM* RNA recognition motif, *G rich* glycine rich motif, *KH* K-homology domain, *dsRBD* double-stranded RNA-binding domain, *Q/G/S/Y* Gln-Gly-Ser-Tyr motif, *ZnF* zinc finger motif, *RGG* Arg-Gly-Gly motif, *G* Gly motif

## Concluding Remarks

In this contribution, we have summarized our current knowledge on the role of RBPs in neurotoxicity. Numerous RBPs are involved in different stages of post-transcriptional control such as alternative splicing, SG dynamics, and mRNA localization. A dysregulation of RBPs to cell stress response at any level may be harmful to neuronal integrity and neuroplasticity. It has been experimentally proven that RBP disorders participate in different pathologies of the CNS and that the main diseases associated with TDP-43 proteinopathy are visualized in motor neuron disorders such as FTD and ALS. The hallmark of RBPs may help elucidate a new perspective involved in neurotoxicity mechanisms. Although some molecular mechanisms associated with RBP functions have been characterized, these are far from being fully understood, elucidating the pathogenesis associated with dysfunctional RBPs and altered local translation could contribute to discovering new drugs that could alleviate the pathology of neurological diseases.

## Abbreviations

4G: GGGGCC repeats
AD: Alzheimer disease
ADAR2: Adenosine deaminase acting on RNA 2
ALCAM: Activated leukocyte cell adhesion molecule
ALS: Amyotrophic lateral sclerosis
APA sites: Alternative polyadenylation sites
APEX2: Apurinic/apyrimidinic endodeoxyribonuclease 2
ARE: Adenylate and uridylate rich elements
ASD: Autism disorder
ASD: Autism spectrum disorder
*CEPB*: Cytoplasmic polyadenylation element-binding protein
CNS: Central nervous system
COPD: Chronic obstructive pulmonary disease
CPE: Cytoplasmic polyadenylation element
CPSF: Cleavage and polyadenylation specificity factor
CstF: Cleavage stimulatory factor
*Cugbp2*: Elav-like family member 2
dsRBD: Double-stranded RNA-binding motif
eIF4F: Eukaryotic initiation factor 4F
eIF4G: Eukaryotic translation initiation factor 4G
ESE: Exonic splicing enhancer
ESS: Exonic splicing silencer
FMRP: Fragile X mental retardation protein
FOX-1: RNA-binding protein Fox-1 Homolog 1
FTD: Frontotemporal lobar dementia
FUS: RNA-binding protein fused in sarcoma
FXS: Fragile X syndrome
G rich: Glycine rich motif
G3BP1: G3BP stress granule assembly factor 1
hnRNPD0: Heterogeneous nuclear ribonucleoprotein D0
hnRNPs: Heterogeneous ribonucleoprotein particles
HNS: Nuclear transport sequence
HuR: Human antigen R
IDR: Intrinsically disordered regions
IPF: Idiopathic pulmonary fibrosis
ISE: Intronic splicing enhancer
ISR: Integrated stress response
ISS: Intronic splicing silencer
ITPR1: Inositol-1,4,5-triphosphate receptor type 1
K rich: Lysine rich motif
KH: K-homology domain
KSRP: K-homology splicing regulatory protein
LCR: Low-complexity regions
MATR3: Matrin 3
MBNL: Muscleblind like splicing regulator 1
MD: Myotonic dystrophy
mRNA: Messenger ribonucleic acid
MSP: Multisystem proteinopathy
Nova: Neuro-oncological ventral antigen
P rich: Proline-rich motif
PABP: Binding of Poly A binding proteins
PARP: Poly ADP-ribose polymerase
PB: P-bodies
PEM: Paraneoplastic encephalomyelitis
PKC: Protein kinase C
POMA: Paraneoplastic myoclonic opsoclonus ataxia
Ptbp1/2: Polypyrimidine tract-binding proteins 1/2
Q/G/S/Y: Gln-Gly-Ser-Tyr motif
R rich: Arginine rich motif
RBD: RNA-binding domains
RBP: RNA-binding proteins
RGG: Arg-Gly-Gly motif
RNA: Ribonucleic acid
RNP: Ribonucleoprotein
ROS: Reactive oxygen species
RRM: RNA recognition motif
S rich: Serine rich motif
SCA2: Spinocerebral ataxia type 2
SG: Stress granules
*Slc8a1*: Solute carrier family 8 member A1
SMA: Spinal muscular atrophy
SR: Serine-arginine proteins
SSN: Subacute sensory neuropathy
TDP-43: TAR DNA-binding protein 43
TIA-1: T-cell internal antigen-1
TIAR: TIA-1 related protein
TTP: Tristetraprolin
UTR: Untranslated regions
Y rich: Tyrosine rich motif
ZBP1: Zip code binding protein 1
ZnF: Zinc finger motif
